# Prognostic Significance of Amino Acid Metabolism-Related Genes in Prostate Cancer Retrieved by Machine Learning

**DOI:** 10.3390/cancers15041309

**Published:** 2023-02-18

**Authors:** Ivana Samaržija, Koraljka Gall Trošelj, Paško Konjevoda

**Affiliations:** Laboratory for Epigenomics, Division of Molecular Medicine, Ruđer Bošković Institute, 10000 Zagreb, Croatia

**Keywords:** prostate cancer, prognosis, progression-free survival, recursive partitioning, Gleason score, CSAD, SERINC3, hypotaurine, phosphatidylserine and sphingolipids

## Abstract

**Simple Summary:**

Prostate cancer is a highly heterogenous disease with respect to molecular, morphological and clinical features. Therefore, one of the major tasks in its management is to define the risk subgroups that would guide the treatment approach. Amino acid metabolism-related genes are involved in several aspects of prostate cancer progression. In this publication, we show that their expression is highly aberrant in prostate cancer, which could be potentially exploited for the establishment of disease progression parameters and therapeutic targets. We show that among the variables studied, the Gleason score was the strongest prognostic factor of progression-free survival in multivariate analysis. Additionally, the expression of *SERINC3* and *CSAD* genes strongly differentiated between better and worse prognosis (low and high risk) for high and low Gleason scores, respectively. These results offer a suggestion for potential biomarkers of prostate cancer progression in patients that are stratified by the Gleason score.

**Abstract:**

Prostate cancer is among the leading cancers according to both incidence and mortality. Due to the high molecular, morphological and clinical heterogeneity, the course of prostate cancer ranges from slow growth that usually does not require immediate therapeutic intervention to aggressive and fatal disease that spreads quickly. However, currently available biomarkers cannot precisely predict the course of a disease, and novel strategies are needed to guide prostate cancer management. Amino acids serve numerous roles in cancers, among which are energy production, building block reservoirs, maintenance of redox homeostasis, epigenetic regulation, immune system modulation and resistance to therapy. In this article, by using The Cancer Genome Atlas (TCGA) data, we found that the expression of amino acid metabolism-related genes is highly aberrant in prostate cancer, which holds potential to be exploited in biomarker design or in treatment strategies. This change in expression is especially evident for catabolism genes and transporters from the solute carrier family. Furthermore, by using recursive partitioning, we confirmed that the Gleason score is strongly prognostic for progression-free survival. However, the expression of the genes *SERINC3* (phosphatidylserine and sphingolipids generation) and *CSAD* (hypotaurine generation) can refine prognosis for high and low Gleason scores, respectively. Therefore, our results hold potential for novel prostate cancer progression biomarkers.

## 1. Introduction

Prostate cancer is among the leading cancers according to both incidence and mortality. It is estimated that in 2020, there were 1,414,259 (7.3% of all sites) new cases diagnosed and 375,304 (3.8% of all sites) deaths from this disease [1]. Common treatment options for confined prostate cancer include surgical removal of the prostate (radical prostatectomy) and radiotherapy. However, biochemical recurrence, defined by a significant rise in blood levels of prostate-specific antigen, occurs within approximately 10 years in 20–40% of patients after radical prostatectomy and 30–50% after radiotherapy [2]. The biochemical recurrence can lead to a progressive disease, which is accompanied by symptoms or evidence of disease progression on imaging [3]. One of the major problems in prostate cancer management is to predict the course of a disease, that is, to differentiate between the tumors that will grow slowly and require minimal or no treatment and those that are more aggressive and will progress fast. Therefore, novel treatment strategies and therapeutic targets are needed, as well as better biomarkers, which would guide prostate cancer management.

Metabolic rewiring is one of the hallmarks of cancer [4], through which the cancer cell satisfies its high demands for energy and biomass building blocks to sustain its rapid proliferation. In comparison to other solid cancer types, which largely rely on aerobic glycolysis (the Warburg effect), prostate cancer cells use oxidative phosphorylation more than non-transformed prostate cells [5,6,7]. However, in advanced stages of prostate cancer, an increased glycolytic phenotype has been observed. In addition to these specificities, a hallmark of the metastatic, castration-resistant prostate cancer (mCRPC) is lipid metabolism rewiring, which manifests as increased fatty acids and cholesterol synthesis, uptake and oxidation [8].

Along with carbohydrates and fatty acids, amino acids are among the main sources of nutrients for energy homeostasis (alternative fuels) and building blocks for macromolecular biosynthesis. Additionally, amino acids help to maintain the redox balance as they are the main elements for reduced glutathione (GSH) and nicotinamide adenine dinucleotide phosphate (NADPH) generation, which are among the key molecules involved in control of the cellular redox state. Amino acid derivatives contribute to epigenetic modifications and posttranscriptional regulation. Namely, one-carbon units from the methionine and folate cycle are methyl donors for DNA and histone methylation, while acetyl-CoA derived from a group of amino acids can be used for histone acetylation. Amino acids also largely influence immune system responses in tumorigenesis and metastasis formation by creating an immunosuppressive or immunoeffective microenvironment [9]. Moreover, amino acids enable cancer cells to circumvent anticancer therapies [10]. The metabolism and uptake of amino acids, therefore, are aberrantly upregulated in many cancer types, and some of those cancer types are characterized by addiction to particular amino acids [11]. For these reasons, amino acid depletion therapies are extensively being explored in the area of cancer research [12].

The amino acid profile in prostate cancer, unlike in other solid tumors, is characterized by their anaplerotic roles more than by energy-production roles. Anaplerotic reactions are chemical reactions that form intermediates of a metabolic pathway and fuel that certain pathway. Many of the amino acids are implicated in prostate cancer, and their involvement has been recently reviewed [13,14]. For example, amino acids commonly related to prostate cancer include glutamine, leucine, serine, glycine, sarcosine, proline and arginine. In the light of the results of this paper, we describe further the roles of serine and taurine in prostate cancer. 

Serine/glycine biosynthesis and one-carbon metabolism are intertwined and essential in promoting cancer cell survival and rapid proliferation. The excessive activation of serine/glycine biosynthesis pathways drives tumorigenesis and provides a single carbon unit for one-carbon metabolism. One-carbon metabolism, which is based on the chemical reactions of methionine and folate compounds, is used for the de novo synthesis of nucleotides, polyamines, amino acids, creatine and phospholipids. Serine is also a precursor for the synthesis of glycine and cysteine, both of which contribute to the production of glutathione, which is essential for redox homeostasis [15,16]. In prostate cancer, it was recently shown that increased serine and one-carbon pathway metabolism promote a neuroendocrine phenotype, which is the most lethal subtype of castration-resistant prostate cancer [17]. This characteristic represents a targetable vulnerability for prostate cancer [18,19]. In line with these findings, the role of alanine-serine-cysteine transporter 2 (ASCT2, SLC1A5) was studied. ASCT2 is a Na+-dependent transporter involved in the cellular uptake of neutral amino acids, that is, amino acids with small, hydrophilic side chains, such as serine, cysteine, asparagine and glutamine, but also alanine with the nonpolar methyl side chain [20]. The inhibition of ASCT2 suppresses prostate cancer cell growth in vitro. However, the contribution of serine to this process was not delineated, and the preferred substrate for ASCT2 is the conditionally essential amino acid glutamine [21,22]. Along with ASCT2 and several other transporters, SERINC3 (Serine Incorporator 3) protein was predicted to enable L-serine transmembrane transporter activity.

Taurine was also suggested to be potentially involved in prostate cancer progression. Namely, taurine was shown to attenuate the expression of epithelial–mesenchymal transition-related genes in human prostate cancer cells [23]. It also promoted apoptosis and inhibited proliferation of the prostate cancer cell line DU145, probably through the MST1/Hippo signaling pathway [24]. In another paper, it was shown that taurine suppressed PSA and metastasis-related genes expression in the human prostate cancer cell lines LNCaP and PC-3. In addition, taurine inhibited the migration of LNCaP and PC-3 cells [25]. Hypotaurine is a sulfinic acid that is an intermediate in the biosynthesis of taurine. An important gene in the metabolism of (hypo)taurine is *CSAD* (cysteine sulfinic acid decarboxylase). Its protein product catalyzes the decarboxylation of L-aspartate, 3-sulfino-L-alanine and L-cysteate to beta-alanine, hypotaurine and taurine, respectively. The preferred CSAD substrate is 3-sulfino-L-alanine.

In this introductory part we aimed to briefly present the global metabolic changes in prostate cancer and to place the changes in specific amino acid metabolism-related genes into this big picture. Furthermore, in a search for biomarkers that could predict the course of prostate cancer, in this article, we analyzed The Cancer Genome Atlas (TCGA) prostate adenocarcinoma (PRAD) dataset for the expression of amino acid metabolism-related genes. We found that their expression is highly aberrant in prostate cancer. By using a machine learning approach, we found that the expression of the genes *CSAD* and *SERINC3* discriminates between better and worse prognosis (low and high risk) for progression-free survival (PFS) of prostate cancer patients when they are stratified according to the Gleason score. In brief, this article aimed at analyzing the expression and the prognostic significance of amino acid metabolism-related genes in prostate cancer. We believe that this publication (a) adds to the big picture of potential metabolic changes in prostate cancer and (b) suggests potential biomarkers for prostate cancer prognosis. Another value of this paper, in our opinion, is methodological, and that is because (c) we used machine learning techniques (recursive partitioning and survival tree) for the definition of prognostic subgroups, unlike many of the scientific papers with a similar topic that used Cox proportional hazards regression analysis for the definition of each gene’s prognostic abilities. Considering the prostate cancer heterogeneity, we believe that our method better captures its complexity.

## 2. Materials and Methods

### 2.1. Data Preparation and Differential Gene Expression Analysis

Amino acid metabolism-related genes were retrieved from The Molecular Signatures Database (MSigDB) [26] by using Gene Ontology Biological Process (GOBP) categories. The genes that were used in our analyses are listed in the Supplementary Table S1. Briefly, the following categories were considered: amino acid activation, homeostasis, transport, salvage, biosynthesis, metabolism, catabolism, response to amino acid starvation and C- and N-terminal protein amino acid modification. The final list contained 518 genes.

The Cancer Genome Atlas [27] prostate adenocarcinoma (PRAD) dataset, containing gene expression data and clinical information for 497 prostate cancer patients and corresponding control (surrounding, non-transformed) tissues for a subset of 52 patients, was downloaded and analyzed using the TCGAbiolinks R package [28,29,30]. To obtain more thorough insight into differentially expressed amino acid metabolism-related genes and to search deeper for their transcriptional changes in prostate cancer in comparison to non-transformed prostate tissue, we chose the threshold of │log2FC│ ≥ 0.585 (│fold change│ ≥ 1.5) and p adjusted < 0.01. The data based on differentially expressed amino acid metabolism-related genes obtained in this way (N = 121) are listed in Supplementary Table S2. The expression represents the value of normalized counts.

The clinical data shown in Table 1 were obtained from cBioPortal [31] and the NCI Genomic Data Commons (GDC, TCGA) portal [32]. In total, there were 493 patients with clinical information (age, Gleason score, TNM stage, information related to residual tumor and radiation therapy) available. The event that we analyzed was progression-free survival (93 patients with this event), since, fortunately, only a smaller subset of patients experienced an event needed for the overall survival calculation. Some variables contained missing data. However, decision trees that we obtained in survival analysis by using recursive partitioning method are not as adversely affected by missing data as traditional statistical methods [33].

### 2.2. Functional Enrichment Analysis

The 121 differentially expressed amino acid metabolism-related genes (DEGs) from Supplementary Table S1 were subjected to a functional enrichment analysis, which was conducted by using the Enrichr web server [34,35]. The top 10 Gene Ontology Molecular Function (MF) and Biological Process (BP) terms are shown in Table 2. Table 3 lists the functional annotation of the solute carrier family genes with differential expression in prostate cancer retrieved from www.genecards.org [36]. Additionally, Table 4 lists the functional annotation of the catabolic genes from the category Cellular amino acid catabolic process (GO:0009063) with differential expression in prostate cancer. The functional information was also retrieved from www.genecards.org [36].

### 2.3. Survival Analysis

Pre-processed and normalized, but un-filtered, TCGA [27] expression data for the amino acid metabolism-related genes were obtained through the TCGAbiolinks R package [28,29,30]. The clinical data were added to expression data, organized in a data matrix and analyzed using the data analysis software R [37], version 4.2.1.

For the survival analysis, we used rpart module [38,39] in the programming language R [37]. rpart stands for Recursive PARTitioning and is the most used application for the construction of survival trees. Survival trees obtained via this method enable visual identification and comparisons of prognostic factors in a simple and straightforward manner [40,41]. The method is insensitive to missing data, in contrast to classical statistical methods, and gives reliable and robust conclusions in most clinical scenarios. The method is described in more detail in our previous publications [42]. Briefly, at the beginning of the analysis, all patients are included and in subsequent steps, they are divided into prognostic subgroups in a survival tree. At the first split (root node), a logical check is performed. If the criterion of that node is met, the left side of the tree is approached; otherwise it is the right. This is repeated at each stage (decision node) until the terminal node is reached. Therefore, a survival tree obtained in this way is composed of decision nodes and terminal nodes (leaves). Each decision node uses a provided variable to subdivide patients into two subgroups with a maximum difference in hazard ratios (HRs). The terminal nodes are reached when no further improvement in subdivision is possible. Patients in the first decision node have hazard ratio of 1. The hazard ratio for patients in each node is expressed in comparison to this value. To avoid overfitting, that is, an extensive fragmentation of the tree for which it would be hard to infer a biological meaning, we set the complexity parameter CP to 0.0373.

### 2.4. Kaplan–Meier Survival Estimate

The difference in survival between patients in terminal nodes was analyzed using a log-rank test and is presented as survival curves based on the Kaplan–Meier survival estimate [43]. This part of the analysis was based on the EZR package [44] in programming language R. Data were considered statistically significant if the p value of the log-rank test was ≤0.05.

## 3. Results

### 3.1. Prostate Cancer Amino Acid Metabolism-Related Gene Expression Appears to Be Highly Aberrant

As elaborated previously, amino acid metabolism-related genes play important roles in prostate cancer. To search for amino acid metabolism-related genes that are specifically changed in prostate cancer, we conducted differential gene expression analysis. The results with thresholds │log2FC│ > 0.585 and p adjusted < 0.01 revealed 4215 differentially expressed genes (DEGs) in total. Among them, there were 121 differentially expressed amino acid metabolism-related genes, which are listed in Supplementary Table S2. The enrichment analysis conducted on those 121 differentially expressed genes (Table 2) showed that the expression of genes involved in amino acid transmembrane transport (mainly of the solute carrier family) is highly perturbed. The functional annotation of the solute carrier family genes listed in Supplementary Table S2 is provided in Table 3.

Table 4 lists the roles of Cellular amino acid catabolic process (GO:0009063) genes from Table 2 for which expression changes were observed in prostate cancer. Some important genes involved, for example, in the synthesis of glycine from serine, such as *SHMT2* (serine hydroxymethyltransferase 2), showed increased expression in tumor tissue. The activity of SHMT2 has been suggested to be the primary source of intracellular glycine. Genes that encode proteins involved in the catabolism of L-lysine (AADAT), valine (ACAD8), glycine (GCSH), phenylalanine/tyrosine (GSTZ1), tryptophan (IDO1, TDO2), L-phenylalanine and L-arginine (IL4I1) and serine and glycine (SDS) also showed increased expression. The genes encoding proteins involved in production of the branched-chain amino acids leucine, isoleucine and valine (BCAT2, BCAT1) were also increased in tumor vs. non-transformed tissue. On the other hand, genes with decreased expression in tumor tissue were *AMT*, which is involved in glycine cleavage system; *GLUL*, which catalyzes the synthesis of glutamine from glutamate and ammonia; *NOS1*, nitric oxide synthase, which synthesizes nitric oxide from L-arginine; *PIPOX*, which is involved in L-lysine catabolic process; and *PRODH*, which catalyzes the first step in proline degradation.

For the genes that we show are involved in prostate cancer prognosis (see further section), *CSAD* had increased expression in prostate cancer (fold-change = 1.61, FDR < 0.001, Supplementary Table S2), while the expression of *SERINC3* did not change according to the criteria used.

### 3.2. CSAD and SERINC3 Genes Further Refine the Prognostic Value of the Gleason Score in Prostate Cancer

**Figure 1:**
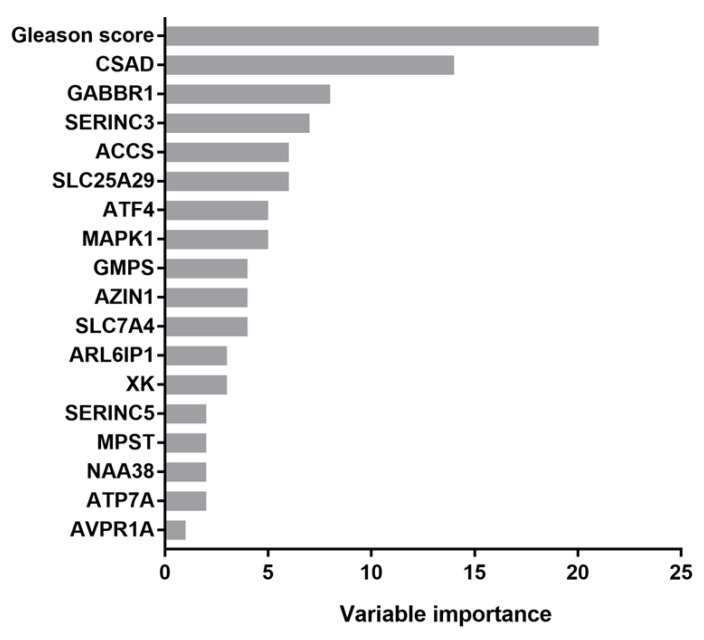
Variable importance determined via the rpart method. CSAD, cysteine sulfinic acid decarboxylase; GABBR1, gamma-aminobutyric acid type B receptor subunit 1; SERINC3, serine incorporator 3; ACCS, 1-aminocyclopropane-1-carboxylate synthase homolog (inactive); SLC25A29, solute carrier family 25 member 29; ATF4, activating transcription factor 4; MAPK1, mitogen-activated protein kinase 1; GMPS, guanine monophosphate synthase; AZIN1, antizyme inhibitor 1; SLC7A4, solute carrier family 7 member 4; ARL6IP1, ADP ribosylation factor-like GTPase 6 interacting protein 1; XK, X-linked Kx blood group antigen, Kell and VPS13A-binding protein; SERINC5, serine incorporator 5; MPST, mercaptopyruvate sulfurtransferase; NAA38, N-alpha-acetyltransferase 38, NatC auxiliary subunit; ATP7A, ATPase copper transporting alpha; AVPR1A, arginine vasopressin receptor 1A.

Prognostic values of variables listed in Table 1 (age, Gleason score, TNM staging, residual tumor information and radiation therapy) supplemented with gene expression data for amino acid metabolism-related genes were determined using recursive partitioning, the recommended method by the AJCC (American Joint Committee on Cancer) for the analysis of prognostic studies [40,41]. The importance of individual variables is shown in Figure 1. The four most informative variables were the Gleason score and the expression of *CSAD*, *GABBR1* and *SERINC3* genes. Among them, only GABBR1 did not appear in multivariate analysis. The *GABBR1* gene encodes a receptor for gamma-aminobutyric acid (GABA), which is the main inhibitory neurotransmitter in the mammalian central nervous system. Its role in the progression of prostate cancer has been documented [45].

**Figure 2:**
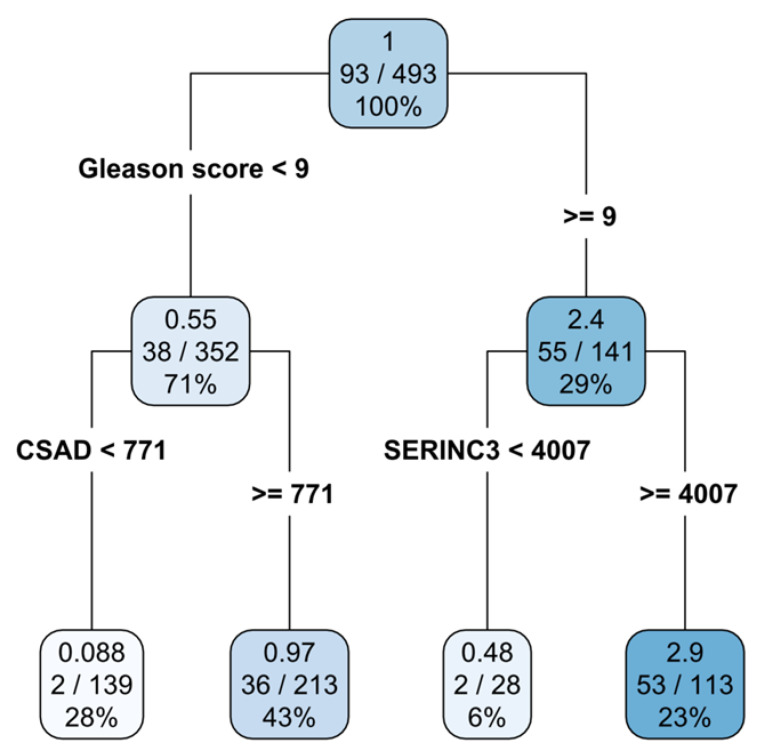
Survival tree constructed using the rpart method identifies four terminal subgroups of patients. The shading of the color denotes the risk group (darker color stands for a higher risk).

However, AJCC criteria for prognostic studies require that a prognostic value of a variable must be always assessed in the context of other variables [40,41]. The rpart algorithm obeys this criterion since rpart uses all variables (multivariate approach) in the analysis. The rpart results are presented on a survival tree (Figure 2). Figure 2 shows that by using three variables, patients could be subdivided into three decision nodes and four terminal nodes (leaves). Variables used in the decision nodes were as follows: (1) the Gleason score, (2) *CSAD* gene expression (for Gleason score < 9), and (3) *SERINC3* gene expression (for Gleason score ≥ 9). The importance of variables was determined by their position in the survival tree: the topmost variable (Gleason score) is the most informative, the variable below topmost is the second one by information value, and so on. The first number in a decision node rectangle denotes the hazard ratio (HR) and the numbers in the second row denote patients with the event (progression) vs. the total number of patients. The number in a third row denotes the percentage of patients in that node. Therefore, it is evident that, while the analysis starts with all patients included in the study (decision node 1; N = 493; N with progression = 93), decision node 2 is based on 71% and decision node 3 on 29% of patients. Further refinement of survival data revealed four prognostic groups: low Gleason score, low *CSAD* expression (28% of patients); low Gleason score, high *CSAD* expression (43%); high Gleason score, low *SERINC3* expression (6%); and finally, high Gleason score, high *SERINC3* expression (23%). The leftmost terminal node represents the group of patients at a very low risk (HR = 0.088), and the second represents patients at a medium risk (HR = 0.97). The second terminal node from the right represent patients at a low risk (HR = 0.48) and the right-most terminal node describes patients at a high risk of prostate cancer progression (HR = 2.9) (Table 5). To emphasize once again, patients in the first decision node have a hazard ratio of 1. The hazard ratio for patients in each node is expressed in comparison to this value. In conclusion, by using the information based on the Gleason score and the expression of *CSAD* and *SERINC3* genes, a subdivision of prostate cancer patients into four prognostic groups with substantially different HRs was achieved.

### 3.3. Kaplan–Meier Estimate on Prostate Cancer Patients Stratified According to Gleason Score and CSAD and SERINC3 Expression

**Figure 3:**
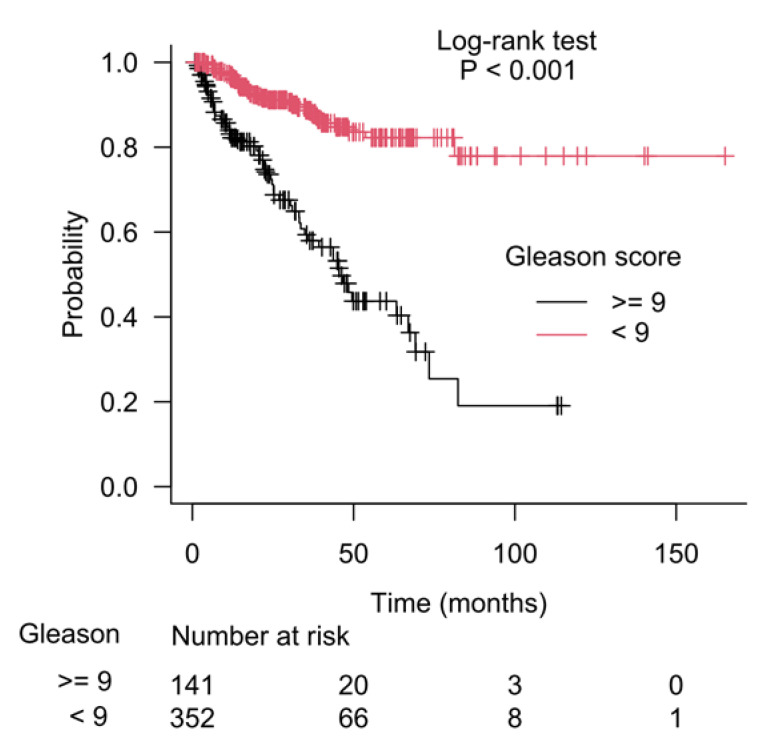
Difference in patients’ survival for the left and the right branches of the starting decision node (node 1), which used the Gleason score as a separation criterion.

**Figure 4:**
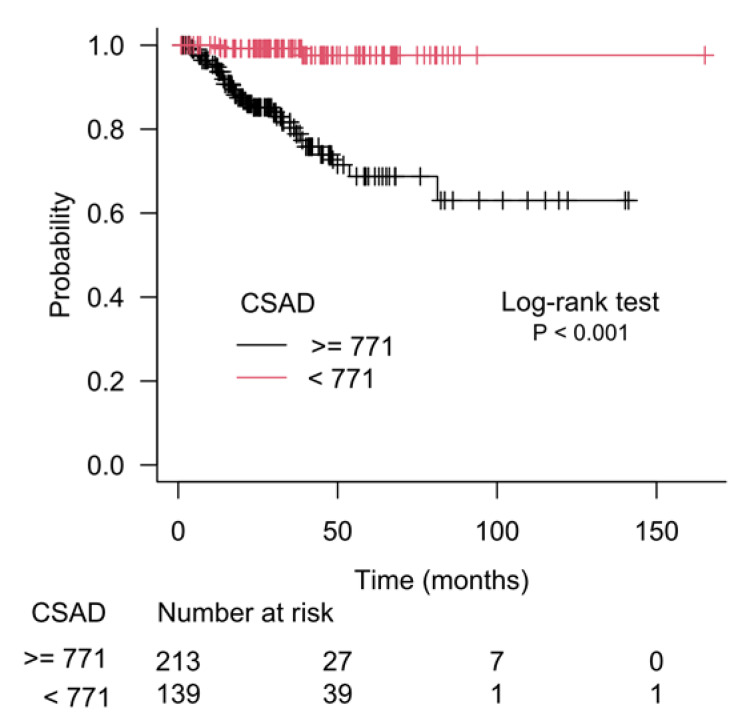
Difference in patients’ survival for the left and the right branches of the second decision node (node 2), which used *CSAD* gene expression as a separation criterion.

**Figure 5:**
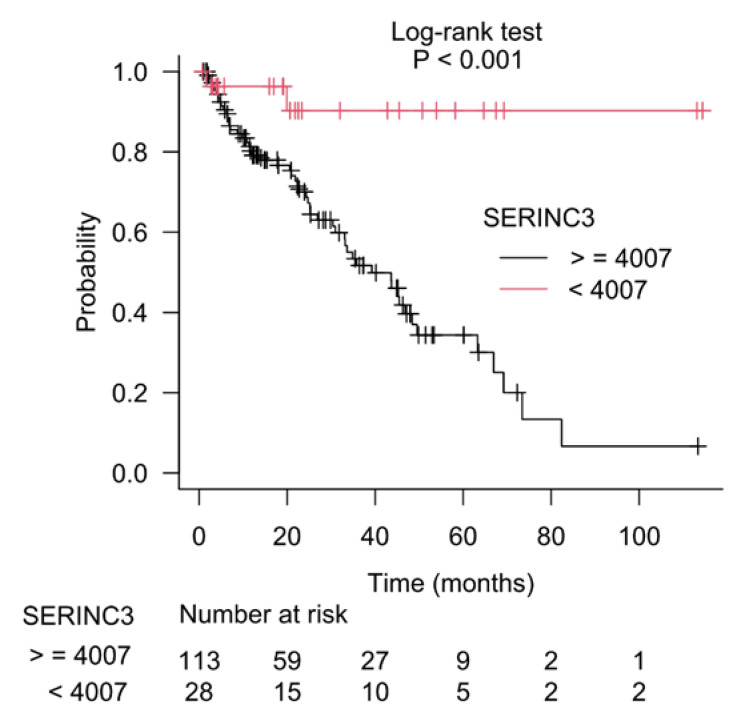
Difference in patients’ survival for the left and the right branches of the third decision node (node 3), which used *SERINC3* gene expression as a separation criterion.

The results of recursive partitioning (Figure 2) were further supplemented by survival curves (Kaplan–Meier method) for subgroups defined in each decision node. The difference for subgroups defined by the left and right branches of decision node 1 is shown in Figure 3, and it was statistically significant (log-rank test, *p* < 0.001). The subgroups defined by the left and right branches of node 2 are shown in Figure 4 (log-rank test, *p* < 0.001). Figure 5 shows that the difference between subgroups of node 3 was also statistically significant (log-rank test, *p* < 0.001).

## 4. Discussion

### 4.1. Metabolites and Metabolism-Related Genes in the Prognosis of Prostate Cancer

The driving events in prostate cancer progression include entangled actions of several signaling pathways that are potentiated by changes in gene expression, genetic and epigenetic alterations [46] and post-transcriptional and post-translational modifications [47]. However, although a substantial amount of information is gathered in regard to the mentioned processes, one of the major obstacles in prostate cancer management is still the inability to predict the course of a disease, that is, to differentiate between slowly growing cancers that do not require immediate treatment and those that are more aggressive and will progress fast.

The metabolic landscape in cancers is highly perturbed in comparison to that in healthy tissue and metabolic genes and molecules, therefore, hold potential to be exploited in a search for disease biomarkers and novel therapeutic targets. This is especially the case since, not only primary tumors, but also metastases from certain tissues (e.g., liver and some other sites [48,49]), acquire changes in metabolism-related gene expression profiles. Metabolic profiles in prostate cancer have been thoroughly studied and reviewed by Kelly et al. [50] who analyzed the articles reporting metabolites in prostate tissue, blood, urine and prostatic secretions. They showed that amino acids are among the most promising metabolic diagnostic biomarkers and biomarkers of tumor aggressiveness. Some amino acids (e.g., glutamine) were also used in terms of predicting disease recurrence [5]. In addition to metabolites themselves, the repertoire of metabolic genes as a source of prostate cancer biomarkers has already been studied. Namely, Zhang et al. identified three metabolism-associated prostate cancer clusters that were characterized by significantly different outcomes in disease-free survival (DFS), clinical stage, stemness index, tumor microenvironment (including stromal and immune cells), presence of DNA mutation (TP53 and SPOP), copy number variation and microsatellite instability [51]. In a further paper, they established metabolism-scores of tumors to predict the prognosis of prostate cancer. This metabolic score was closely related to the tumor microenvironment, presence of DNA mutations and drug sensitivity [52]. Feng et al. studied energy metabolism-related genes in prostate cancer and defined an energy metabolism-related gene prognostic index, which proved to predict biochemical recurrence for patients with prostate cancer that were undergoing radical prostatectomy [53]. Finally, Zhao et al. were able to predict biochemical-recurrence-free survival (BRFS) using a three-metabolic-gene risk score model in prostate cancer patients [2].

### 4.2. Differentially Expressed Amino Acid Metabolism-Related Genes in Prostate Cancer

Although, as elaborated, several papers already dealt with the potential of metabolic genes in predicting the outcome of prostate cancer patients, none of them, to the best of our knowledge, analyzed the amino-acid metabolism-related genes separately. Since amino acids themselves, as already mentioned [50], are involved in the prognosis for prostate cancer patients, it is to be expected that the genes encoding proteins that participate in their metabolism would also show prognostic capabilities. In very recent papers, the amino acid metabolism genes already showed good performance in the prognosis of e.g., colorectal cancer [54], hepatocellular carcinoma [55], clear cell renal cell carcinoma [56], glioma [57] and head and neck squamous cell carcinomas [58]. In this research, we studied the potential of amino acid metabolism-related genes to predict progression-free survival (PFS) using The Cancer Genome Atlas prostate adenocarcinoma (PRAD) dataset. 

The first relevant finding of this paper is that the expression of the genes encoding proteins that are involved in amino acid transport across both the cellular (majority) and the mitochondrial (to a lesser extent) membrane show changed expression. Namely, the solute carrier (SLC) family genes were among the top terms in functional enrichment analysis of both Gene Ontology (GO) Molecular Function and GO Biological Process categories of differentially expressed genes (DEGs) (Table 2 and Table 3). The SLC group of membrane transport proteins include over 400 members organized into 66 families. Solutes that are transported by the various SLC proteins are extremely diverse and include charged and uncharged organic molecules, inorganic ions and the gas ammonia. However, most of the SLC group members listed in Table 3 are involved in amino acid transport as they were selected because of their connection with amino acid metabolism. Although more of the SLCs are up-regulated (15) than down-regulated (10) in prostate cancer, it is hard to speculate about the ‘big picture’, that is, to establish which of the amino acids are largely influenced by these changes in the expression of SLCs. What is known is that some of these gene products were shown to be implicated in prostate cancer progression, such as, for example, SLC7A5 [59], SLC7A11 [60], SLC11A1 [61], SLC43A1 [62] and SLC1A3 [63]. Although not listed in the Table 3, a recent paper documented metabolic reprogramming and the predominance of several solute carrier genes (*SLC12A5*, *SLC25A17* and *SLC27A6*) during acquired enzalutamide resistance in prostate cancer [64], emphasizing the importance of the SLC family members in prostate cancer. 

Another group of genes with changed expression in prostate cancer includes the genes coding for proteins that are involved in the catabolism of different amino acids, as elaborated in the Results section and shown in Table 2 and Table 4.

### 4.3. Prognostic Value of Amino Acid Metabolism-Related Genes in Prostate Cancer

To get back to the primary question of this publication, which would be the prediction of prostate cancer outcomes, several publications already used gene expression profiles to foresee the prostate cancer prognosis (e.g., [65,66,67,68,69,70,71,72]). However, as already mentioned, those still did not make it to the clinics; that is, the course of prostate cancer remains mainly unpredictable. Therefore, in this paper, we extended the knowledge on potential prostate cancer progression-free survival biomarkers to amino acid metabolism-related genes. The changes in expression of those genes are extensive in prostate cancer and therefore hold potential for biomarkers and therapeutic targets. We found that the Gleason score is the strongest variable influencing prostate cancer progression-free survival in a multivariate analysis. This is to be expected, since the Gleason score is highly informative of the characteristics of tumor cells that constitute the tumor tissue. However, when the patients were stratified according to a low/high Gleason score, the genes *CSAD* (for the low Gleason score) and *SERINC3* (for the high Gleason score) differentiated the risk of progression. That is, patients with higher *CSAD* and higher *SERINC3* expression are at a higher risk of progression (Figure 2).

CSAD protein is involved in the generation of beta-alanine, hypotaurine and taurine. Although papers suggest that taurine has a beneficial role in prostate cancer (see Introduction), it needs to be emphasized that hypotaurine is the preferential product of the biochemical reaction involving CSAD. It was shown that hypotaurine potentiates a malignant phenotype in glioma through aberrant hypoxic signaling. The authors show that taurine, the oxidation metabolite of hypotaurine, decreased intracellular hypotaurine and resulted in glioma cell growth arrest [73]. Therefore, the ratio of hypotaurine/taurine could play a role in prostate cancer as well. Additionally, long non-coding RNA TUG1 (taurine up-regulated 1) was originally identified in a genomic screen of taurine-treated mouse retinal cells [74]. TUG1 accelerates prostate cancer progression [75,76]. Its knockdown inhibits the tumorigenesis and progression of prostate cancer in vitro and in vivo [77] and enhances radiosensitivity [78]. Finally, high expression of TUG1 correlates with progression of the disease and less favorable survival profiles in prostate cancer patients [79]. To emphasize that CSAD plays versatile roles in different cancer types, data from The Human Protein Atlas [80,81] state that *CSAD* is an unfavorable prognostic marker in renal and colorectal cancer, which would agree with our study. However, it is favorable in urothelial, liver, pancreatic and head and neck cancer. To add more complexity to the potential mechanisms of action involving hypotaurine/taurine, CSAD also catalyzes the generation of beta-alanine. It would be interesting to further detangle these complex relationships (hypotaurine–taurine–beta–alanine), of which taurine is the most studied, and define their impact on prostate cancer.

As elaborated in an introductory part, serine metabolism potentiates the malignancy of prostate cancer. The serine incorporator (SERINC) proteins are a family of multipass transmembrane proteins associated with the biosynthesis of serine-containing phospholipids and sphingolipids [82]. More precisely, SERINC2–4 are carrier proteins that incorporate the polar amino acid serine into membranes to facilitate the synthesis of phosphatidylserine and sphingolipids [83]. SERINC proteins were most studied in the context of viral infections during which they are constitutive host resistance factors, which suppress viral infection by incorporating into virus particles [83]. Phosphatidylserine (PS) is a serine-containing phospholipid and a component of the cell membrane. It plays a key role in cell cycle signaling, specifically in relation to apoptosis. Studies using pre-clinical models of prostate cancer showed that antibody-mediated PS blockade reprograms the innate immune system to promote anti-tumor responses. Therefore, bavituximab, a PS-targeting antibody, is being assessed in multiple clinical trials, including those for prostate cancer [84]. Sphingolipids are synthesized from serine and palmitoyl-CoA. Inhibitors of sphingolipid metabolism were shown to antagonize pro-survival responses. Moreover, cancer cells use sphingolipid-driven escape mechanisms to evade therapies. Sphingolipids have also been implicated in prostate cancer, as recently reviewed [85]. This brief overview of the promoting roles of phosphatidylserine and sphingolipids in prostate tumorigenesis agrees with our findings that the increased expression of *SERINC3*, which potentiates their biosynthesis, represents a higher risk of disease progression for prostate cancer patients that are stratified according to the Gleason score. In addition to *SERINC3*, *SERINC5*, for which the gene product has a similar function to SERINC3, is present on the list of genes implicated by our univariate analysis (Figure 1). This further indicates that the processes conducted by proteins encoded by these genes are potentially critically involved in prostate tumorigenesis.

### 4.4. Methodological Considerations

Besides dealing with biological processes involved in prostate cancer progression, our paper differs from those with a similar topic in that we used machine learning to define prognostic subgroups instead of using Cox proportional hazards regression analysis to define gene-based prognosis. From the technical point of view, the recursive partitioning method used has the advantage in that it establishes the hierarchy of the variables studied; that is, this method lists the variables by their importance for prognosis. In this way, subgroups of patients are defined, and the knowledge on their specificities is refined. Given the heterogeneity of prostate cancer, we believe that this method is more suitable to define gene expression-specific prostate cancer characteristics. Additionally, the survival tree, generated through recursive partitioning, is easier to interpret than the Cox regression results.

## 5. Conclusions

In our study, we analyzed differentially expressed genes between prostate cancer and surrounding non-transformed prostate tissue by using TCGA data. We found that the expression of amino acid metabolism-related genes is highly aberrant in prostate cancer. The groups of genes that are the most affected include solute carrier family of amino acid transporters and the genes involved in the catabolism of amino acids, which are mainly up-regulated. Furthermore, we found that the Gleason score is the strongest prognostic factor for progression-free survival in prostate cancer patients, which is expected given the amount of information provided by this parameter. However, when the patients are stratified according to the Gleason score, the genes *CSAD* (low Gleason score) and *SERINC3* (high Gleason score) further refine the prognosis. The high expression of both *CSAD* and *SERINC3* is correlated with worse outcomes. The *CSAD* gene product is involved in hypotaurine generation, and the *SERINC3* gene product is involved in the generation of phosphatidylserine and sphingolipids. There are indications that hypotaurine, phosphatidylserine and sphingolipids promote prostate cancer progression. We believe that our results hold potential for the future design of prognostic biomarkers in prostate cancer, which is an intensive field of research, considering that the progression of prostate cancer is currently hard to predict. Functional studies on *CSAD* and *SERINC3* genes and their regulators are needed to further delineate their roles in prostate cancer, which would reveal their potential for further interventions.

## Figures and Tables

**Table 1:** Clinical information of TCGA patients. The number (N) and the percentage (in parenthesis) of patients belonging to a certain category is shown. In some categories, there are unknowns (NAs).

		No Progression	Progression
N, total		400	93
Age, years	<60	166 (41.5%)	34 (36.6%)
	≥60	234 (58.5%)	59 (63.4%)
Gleason score	6	44 (11%)	1 (1.1%)
	7	221 (55.3%)	24 (25.8%)
	8	49 (12.3)	13 (14%)
	9	84 (21%)	53 (57%)
	10	2 (0.5%)	2 (2.2%)
Clinical T stage	cT1	158 (39.5%)	17 (18.3%)
	cT2	137 (34.3%)	35 (37.6%)
	cT3	28 (7%)	24 (25.8%)
	cT4	1 (0.3%)	1 (1.1%)
	NA	76 (19%)	16 (17.2%)
Clinical M stage	cM0	362 (90.5%)	89 (95.7%)
	cM1	2 (0.5%)	1 (1.1%)
	NA	36 (9%)	3 (3.2%)
Pathologic T stage	pT2	172 (43%)	14 (15.1%)
	pT3	215 (53.8%)	75 (80.7%)
	pT4	7 (1.8%)	3 (3.2%)
	NA	6 (1.5%)	1 (1.1%)
Pathologic N stage	pN0	280 (70%)	62 (66.7%)
	pN1	56 (14%)	22 (23.7%)
	NA	64 (16%)	9 (9.7%)
Residual tumor	R0	266 (66.5%)	46 (49.5%)
	R1	102 (25.5%)	44 (47.3%)
	R2	5 (1.3%)	0
	RX	13 (3.3%)	2 (2.2%)
	NA	14 (3.5%)	1 (1.1%)
Radiation therapy	Yes	48 (12%)	46 (49.5%)
	No	313 (78.3%)	43 (46.2%)
	NA	39 (9.8%)	4 (4.3%)

**Table 2:** Enrichment analysis for the differentially expressed amino acid metabolism-related genes (N = 121). The gene ontology (GO) Molecular Function (MF) and Biological Process (BP) categories are listed.

GO Molecular Function (First 10 Terms) and Biological Process (Last 10 Terms) Categories	Overlap	*p*-Value	Adj. *p*-Value	Genes
Amino acid transmembrane transporter activity (GO:0015171)	16/49	3.26 × 10^−24^	6.98 × 10^−22^	*SLC36A1; SLC6A19; SLC38A1; SLC47A1; SLC43A1; SLC3A1; SLC38A11; SLC7A11; SLC6A1; SLC7A1; SLC7A4; SLC7A5; SLC6A6; PDPN; SLC16A2; SLC38A5*
L-amino acid transmembrane transporter activity (GO:0015179)	13/53	5.15 × 10^−18^	5.51 × 10^−16^	*SLC36A1; SLC38A1; SLC47A1; SLC43A1; SLC1A3; SLC3A1; SLC7A11; SLC7A1; SLC7A5; SLC25A15; SLC25A12; SLC25A22; SLC38A5*
Organic anion transmembrane transporter activity (GO:0008514)	17/144	1.67 × 10^−17^	1.19 × 10^−15^	*SLC36A1; SLC38A1; SLC1A3; SLC3A1; SLC6A1; SLC7A1; SLC6A6; SLC25A15; SLC7A5; GJA1; PDPN; SFXN3; SLC25A21; SFXN2; SLC25A12; SLC25A22; SLC38A5*
Carboxylic acid transmembrane transporter activity (GO:0046943)	12/57	7.73 × 10^−16^	4.14 × 10^−14^	*SLC36A1; SLC7A4; SLC7A5; SLC6A6; SLC38A1; PDPN; SLC3A1; SLC6A11; SLC38A11; SLC16A2; SLC7A1; SLC38A5*
Neutral amino acid transmembrane transporter activity (GO:0015175)	9/32	2.00 × 10^−13^	8.57 × 10^−12^	*SLC36A1; SLC6A6; SLC7A5; SLC6A19; SLC38A1; SLC43A1; SFXN3; SFXN2; SLC38A5*
Cation transmembrane transporter activity (GO:0008324)	9/48	1.10 × 10^−11^	3.94 × 10^−10^	*SLC36A1; SLC6A6; SLC7A5; SLC25A15; SLC38A1; SFXN3; SFXN2; SLC7A1; SLC38A5*
Pyridoxal phosphate binding (GO:0030170)	6/21	2.18 × 10^−9^	6.66 × 10^−8^	*SDS; OAT; SHMT2; CBS; PSAT1; ACCS*
Amino acid: sodium symporter activity (GO:0005283)	5/12	3.35 × 10^−9^	7.96 × 10^−8^	*SLC38A1; SLC6A15; SLC1A3; SLC6A11; SLC6A1*
Transaminase activity (GO:0008483)	5/12	3.35 × 10^−9^	7.96 × 10^−8^	*OAT; AADAT; PSAT1; BCAT1; BCAT2*
Amino acid binding (GO:0016597)	6/32	3.45 × 10^−8^	7.38 × 10^−7^	*GRM7; SHMT2; NOS1; NAGS; ASS1; GNMT*
Cellular amino acid catabolic process (GO:0009063)	25/90	2.11 × 10^−35^	2.40 × 10^−32^	*SHMT2; HAAO; SDSL; DDO; GCSH; IL4I1; TDO2; CBS; SLC25A21; NOS1; PRODH; GLUL; HMGCLL1; ACAD8; MCCC2; SDS; AADAT; GAD1; AMT; PIPOX; GSTZ1; BCAT1; ASPA; IDO1; BCAT2*
Alpha-amino acid metabolic process (GO:1901605)	16/46	9.80 × 10^−25^	5.57 × 10^−22^	*OAT; AADAT; FOLH1B; ASNS; PYCR1; ASS1; GNMT; FOLH1; CPS1; CBS; NOX4; DPEP1; RIMKLA; SLC25A12; GLUL; ASPA*
Amino acid transport (GO:0006865)	16/50	4.77 × 10^−24^	1.81 × 10^−21^	*SLC36A1; SLC6A19; SLC38A1; SLC6A17; SLC6A15; SLC43A1; SLC3A1; SLC38A11; SLC16A10; SLC7A11; SLC7A1; SLC7A4; SLC7A5; SLC6A6; PDPN; SLC38A5*
Amino acid transmembrane transport (GO:0003333)	14/45	5.77 × 10^−21^	1.64 × 10^−18^	*SLC36A1; SLC38A1; SLC47A1; SLC38A11; SLC7A11; SLC7A1; SLC6A6; SLC7A5; SFXN3; SFXN2; SLC16A2; SLC25A12; SLC25A22; SLC38A5*
Amino acid import (GO:0043090)	10/22	2.73 × 10^−17^	6.21 × 10^−15^	*SLC36A1; SLC6A6; SLC7A5; SLC47A1; SFXN3; SLC1A3; SFXN2; SLC6A1; SLC16A2; SLC7A1*
Glutamine family amino acid metabolic process (GO:0009064)	11/37	1.87 × 10^−16^	3.54 × 10^−14^	*OAT; GLYATL1; CPS1; AADAT; PYCR1; NAGS; PRODH; RIMKLA; GLUL; NIT2; ART4*
Nitrogen compound transport (GO:0071705)	15/143	8.73 × 10^−15^	1.42 × 10^−12^	*SLC36A1; SLC6A19; SLC38A1; SLC11A1; SLC6A15; SLC43A1; SLC3A1; SLC16A10; SLC7A11; SLC7A1; SLC7A4; SLC6A6; SLC7A5; PDPN; SLC38A5*
Organic acid transport (GO:0015849)	13/100	3.43 × 10^−14^	4.88 × 10^−12^	*SLC36A1; SLC6A19; SLC38A1; SLC6A15; SLC43A1; SLC3A1; SLC16A10; SLC7A11; SLC7A1; SLC7A4; SLC6A6; PDPN; SLC38A5*
Import into cell (GO:0098657)	10/41	4.3 × 10^−14^	5.44 × 10^−12^	*SLC36A1; SLC6A6; SLC7A5; SLC38A1; SLC47A1; SLC1A3; ATP1A2; SLC16A2; SLC7A1; GLUL*
Aspartate family amino acid metabolic process (GO:0009066)	9/30	1.01 × 10^−13^	1.07 × 10^−11^	*FOLH1; FOLH1B; SMS; ASNS; SLC25A12; ASPA; NIT2; ASS1*

**Table 3:** Functional annotation of solute carrier (SLC) family genes for which expression changes were observed in prostate cancer. The up- and down-regulated genes are listed separately. FC indicates fold change (tumor, T vs. normal, N), and FDR is the false discovery rate.

Gene	Function	FC (T/N)	FDR
*SLC3A1*	Transports neutral and basic amino acids in the renal tubule and intestinal tract.	2.72	2.93 × 10^−5^
*SLC6A11*	Sodium-dependent transporter that uptakes gamma-aminobutyric acid (GABA), an inhibitory neurotransmitter, which ends the GABA neurotransmission.	3.72	7.57 × 10^−13^
*SLC6A15*	Encodes a member of the solute carrier family 6 protein family, which transports neutral amino acids.	2.15	0.003273
*SLC6A17*	Responsible for the presynaptic uptake of neurotransmitters. The encoded vesicular transporter is selective for proline, glycine, leucine and alanine.	3.61	8.27 × 10^−10^
*SLC6A19*	Encodes a system B(0) transmembrane protein that actively transports most neutral amino acids across the apical membrane of epithelial cells.	6.40	0.000127
*SLC7A1*	Enables L-arginine transmembrane transporter activity and L-histidine transmembrane transporter activity.	1.52	9.93 × 10^−8^
*SLC7A11*	Encodes a member of a heteromeric, sodium-independent, anionic amino acid transport system that is highly specific for cysteine and glutamate.	3.67	6.95 × 10^−22^
*SLC11A1*	Member of the proton-coupled divalent metal ion transporters family; encodes a multi-pass membrane protein that functions as a divalent transition metal (iron and manganese) transporter involved in iron metabolism.	1.79	2.91 × 10^−11^
*SLC16A10*	Member of a family of plasma membrane amino acid transporters that mediate the Na(+)-independent transport of aromatic amino acids across the plasma membrane.	1.56	0.000213
*SLC25A15*	Member of the mitochondrial carrier family. The encoded protein transports ornithine across the inner mitochondrial membrane from the cytosol to the mitochondrial matrix. The protein is an essential component of the urea cycle and functions in ammonium detoxification and biosynthesis of the amino acid arginine.	1.74	3.49 × 10^−14^
*SLC25A21*	Mitochondrial carrier that transports C5-C7 oxodicarboxylates across inner mitochondrial membranes.	1.98	5.81 × 10^−12^
*SLC25A22*	Encodes a mitochondrial glutamate carrier.	1.88	1.43 × 10^−24^
*SLC36A1*	The encoded protein functions as a proton-dependent, small amino acid transporter.	1.80	3.82 × 10^−6^
*SLC38A11*	Predicted to enable amino acid transmembrane transporter activity.	2.45	1.02 × 10^−6^
*SLC43A1*	Belongs to the system L family of plasma membrane carrier proteins that transports large neutral amino acids.	2.72	2.92 × 10^−17^
*SLC1A3*	Member of a high affinity glutamate transporter family.	0.55	1.63 × 10^−12^
*SLC6A1*	The protein encoded by this gene is a gamma-aminobutyric acid (GABA) transporter that localizes to the plasma membrane.	0.65	3.31 × 10^−5^
*SLC6A6*	This gene encodes a multi-pass membrane protein that is a member of a family of sodium and chloride-ion-dependent transporters. The encoded protein transports taurine and beta-alanine.	0.64	1.32 × 10^−9^
*SLC7A4*	Predicted to enable amino acid transmembrane transporter activity. Predicted to be involved in amino acid transport.	0.53	0.001077
*SLC7A5*	Enables L-leucine transmembrane transporter activity, L-tryptophan transmembrane transporter activity and thyroid hormone transmembrane transporter activity.	0.31	2.00 × 10^−26^
*SLC16A2*	Encodes an integral membrane protein that functions as a transporter of thyroid hormone.	0.58	1.00 × 10^−17^
*SLC25A12*	Encodes a calcium-binding mitochondrial carrier protein. The encoded protein localizes to the mitochondria and is involved in the exchange of aspartate for glutamate across the inner mitochondrial membrane.	0.64	3.29 × 10^−24^
*SLC38A1*	An important transporter of glutamine, an intermediate in the detoxification of ammonia and the production of urea.	0.64	9.92 × 10^−14^
*SLC38A5*	The encoded protein transports glutamine, asparagine, histidine, serine, alanine and glycine across the cell membrane, but does not transport charged amino acids, imino acids, or N-alkylated amino acids.	0.45	3.30 × 10^−16^
*SLC47A1*	Among its related pathways are the transport of inorganic cations/anions and amino acids/oligopeptides.	0.39	6.09 × 10^−29^

**Table 4:** Functional annotation of Cellular amino acid catabolic process (GO:0009063) genes from Table 2 for which expression changes were observed in prostate cancer. The up- and down-regulated genes are listed separately. FC indicates fold change (tumor, T vs. normal, N), and FDR is the false discovery rate.

Gene	Function	FC (T/N)	FDR
*AADAT*	Aminoadipate aminotransferase. Highly similar to mouse and rat kynurenine aminotransferase II. The rat protein is a homodimer with two transaminase activities. One activity is the transamination of alpha-aminoadipic acid, a final step in the saccaropine pathway, which is the major pathway for L-lysine catabolism. The other activity involves the transamination of kynurenine to produce kynurenine acid, the precursor of kynurenic acid.	2.01	6.04 × 10^−12^
*ACAD8*	Acyl-CoA dehydrogenase family member 8. This gene encodes a member of the acyl-CoA dehydrogenase family of enzymes that catalyzes the dehydrogenation of acyl-CoA derivatives in the metabolism of fatty acids or branch-chained amino acids. The encoded protein is a mitochondrial enzyme that functions in catabolism of the branched-chain amino acid valine.	1.67	2.96 × 10^−5^
*BCAT1*	Branched chain amino acid transaminase 1. This gene encodes the cytosolic form of the enzyme branched-chain amino acid transaminase. This enzyme catalyzes the reversible transamination of branched-chain alpha-keto acids to branched-chain L-amino acids essential for cell growth.	1.71	0.00045
*BCAT2*	Branched chain amino acid transaminase 2. This gene encodes a branched-chain aminotransferase found in mitochondria. The encoded protein forms a dimer that catalyzes the first step in the production of the branched-chain amino acids leucine, isoleucine and valine.	1.51	3.01 × 10^−12^
*CBS*	Cystathionine beta-synthase. The protein encoded by this gene acts as a homotetramer to catalyze the conversion of homocysteine to cystathionine, the first step in the transsulfuration pathway.	2.23	2.09 × 10^−14^
*GAD1*	Glutamate decarboxylase 1. This gene encodes one of several forms of glutamic acid decarboxylase, identified as a major autoantigen in insulin-dependent diabetes. The enzyme encoded is responsible for catalyzing the production of gamma-aminobutyric acid from L-glutamic acid.	3.07	4.07 × 10^−13^
*GCSH*	Glycine cleavage system protein H. The degradation of glycine is brought about by the glycine cleavage system, which is composed of four mitochondrial protein components: P protein (a pyridoxal phosphate-dependent glycine decarboxylase), H protein (a lipoic acid-containing protein), T protein (a tetrahydrofolate-requiring enzyme), and L protein (a lipoamide dehydrogenase). The protein encoded by this gene is the H protein, which transfers the methylamine group of glycine from the P protein to the T protein.	1.62	1.98 × 10^−5^
*GSTZ1*	Glutathione S-transferase zeta 1. This gene is a member of the glutathione S-transferase (GST) super-family that encodes multifunctional enzymes important in the detoxification of electrophilic molecules, including carcinogens, mutagens and several therapeutic drugs, via conjugation with glutathione. This enzyme catalyzes the conversion of maleylacetoacetate to fumarylacetoacatate, which is one of the steps in the phenylalanine/tyrosine degradation pathway.	1.51	1.98 × 10^−9^
*IDO1*	Indoleamine 2,3-dioxygenase 1. This gene encodes indoleamine 2,3-dioxygenase (IDO)—a heme enzyme that catalyzes the first and rate-limiting step in tryptophan catabolism to N-formyl-kynurenine. This enzyme acts on multiple tryptophan substrates, including D-tryptophan, L-tryptophan, 5-hydroxy-tryptophan, tryptamine, and serotonin.	1.51	0.009556
*IL4I1*	Interleukin 4 induced 1. This gene encodes a secreted L-amino acid oxidase protein, which primarily catabolizes L-phenylalanine and, to a lesser extent, L-arginine.	1.81	5.50 × 10^−10^
*MCCC2*	Methylcrotonyl-CoA carboxylase subunit 2. This gene encodes the small subunit of 3-methylcrotonyl-CoA carboxylase. This enzyme functions as a heterodimer and catalyzes the carboxylation of 3-methylcrotonyl-CoA to form 3-methylglutaconyl-CoA.	2.45	5.63 × 10^−12^
*SDS*	Serine dehydratase. This gene encodes one of three enzymes that are involved in metabolizing serine and glycine. L-serine dehydratase converts L-serine to pyruvate and ammonia and requires pyridoxal phosphate as a cofactor. The encoded protein can also metabolize threonine to NH4+ and 2-ketobutyrate.	3.32	1.36 × 10^−14^
*SDSL*	Serine dehydratase like. Predicted to be involved in the isoleucine biosynthetic process and threonine catabolic process.	1.52	3.11 × 10^−10^
*SHMT2*	Serine hydroxymethyltransferase 2. This gene encodes the mitochondrial form of a pyridoxal phosphate-dependent enzyme that catalyzes the reversible reaction of serine and tetrahydrofolate to glycine and 5,10-methylene tetrahydrofolate. The encoded product is primarily responsible for glycine synthesis. The activity of the encoded protein has been suggested to be the primary source of intracellular glycine.	1.69	7.99 × 10^−17^
*SLC25A21*	Solute carrier family 25 member 21. Homolog of the S. cerevisiae ODC proteins, mitochondrial carriers that transport C5-C7 oxodicarboxylates across inner mitochondrial membranes. One of the species transported by ODC is 2-oxoadipate, a common intermediate in the catabolism of lysine, tryptophan and hydroxylysine in mammals.	1.98	5.81 × 10^−12^
*TDO2*	Tryptophan 2,3-dioxygenase. This gene encodes a heme enzyme that plays a critical role in tryptophan metabolism by catalyzing the first and rate-limiting step of the kynurenine pathway.	3.45	0.0044
*AMT*	Aminomethyltransferase. This gene encodes one of four critical components of the glycine cleavage system.	0.52	7.11 × 10^−13^
*ASPA*	Aspartoacylase. This gene encodes an enzyme that catalyzes the conversion of N-acetyl-L-aspartic acid (NAA) to aspartate and acetate.	0.24	6.87 × 10^−31^
*DDO*	D-aspartate oxidase. The protein encoded by this gene is a peroxisomal flavoprotein that catalyzes the oxidative deamination of D-aspartate and N-methyl D-aspartate.	0.50	1.01 × 10^−15^
*GLUL*	Glutamate-ammonia ligase. The protein encoded by this gene belongs to the glutamine synthetase family. It catalyzes the synthesis of glutamine from glutamate and ammonia in an ATP-dependent reaction.	0.64	7.49 × 10^−16^
*HAAO*	3-Hydroxyanthranilate 3,4-dioxygenase is a monomeric cytosolic protein belonging to the family of intramolecular dioxygenases containing nonheme ferrous iron. HAAO catalyzes the synthesis of quinolinic acid (QUIN) from 3-hydroxyanthranilic acid.	0.45	2.36 × 10^−19^
*HMGCLL1*	3-Hydroxymethyl-3-methylglutaryl-CoA lyase like 1. Non-mitochondrial 3-hydroxymethyl-3-methylglutaryl-CoA lyase that catalyzes the cation-dependent cleavage of (S)-3-hydroxy-3-methylglutaryl-CoA into acetyl-CoA and acetoacetate, a key step in ketogenesis.	0.32	5.18 × 10^−15^
*NOS1*	Nitric oxide synthase 1. The protein encoded by this gene belongs to the family of nitric oxide synthases, which synthesize nitric oxide from L-arginine.	0.28	2.12 × 10^−14^
*PIPOX*	Pipecolic acid and sarcosine oxidase. Enables L-pipecolate oxidase activity and sarcosine oxidase activity. Involved in L-lysine catabolic process to acetyl-CoA via L-pipecolate.	0.39	3.84 × 10^−24^
*PRODH*	Proline dehydrogenase 1. This gene encodes a mitochondrial protein that catalyzes the first step in proline degradation.	0.35	1.70 × 10^−24^

**Table 5:** Risk subgroups extracted via rpart analysis.

Risk Subgroup	Hazard Ratio	Rule
Very low risk	0.088	Gleason score < 9 AND *CSAD* < 771
Low risk	0.480	Gleason score ≥ 9 AND *SERINC3* < 4007
Medium risk	0.974	Gleason score < 9 AND *CSAD* ≥ 771
High risk	2.923	Gleason score ≥ 9 AND *SERINC3* ≥ 4007

## Data Availability

In this article, we used The Cancer Genome Atlas (TCGA) prostate adenocarcinoma (PRAD) dataset available at https://gdc.cancer.gov/ (accessed on 1 November 2022).

## Supplementary Materials

The following supporting information can be downloaded at: https://www.mdpi.com/article/10.3390/cancers15041309/s1, Table S1: Human amino-acid metabolism-related genes categorized through Gene Ontology Biological Processes (GOBPs; retrieved from https://www.gsea-msigdb.org/gsea/msigdb/index.jsp (accessed on 1 November 2022)); Table S2: Differentially expressed amino-acid metabolism-related genes between prostate cancer tumor and non-tumor tissue with a threshold |log2FC| ≥ 0.585 and FDR < 0.01.
