# Supplementary material for: Prognostic Significance of Amino Acid Metabolism-Related Genes in Prostate Cancer Retrieved by Machine Learning
Source: Cancers (Basel). 2023 Feb 18;15(4):1309. doi: 10.3390/cancers15041309 (PMC9954451; doi:10.3390/cancers15041309)
Supplement: Supplementary file 1 [file cancers-15-01309-s001.zip › cancers-2158349-supplementary.pdf]

cancers MDPI

Table S1. Human amino-acid metabolism-related genes categorized through Gene Ontology Biological Process (GOBP; retrieved from https://www.gsea-msigdb.org/gsea/msigdb/index.jsp).

GO BP Pathway	Genes
AMINO ACID ACTIVATION (GO:0043038)	FARSB, WARS2, FARS2, TARS3, EARS2, AASDH, AARS1, DARS1, EPRS1, FARSA, LARS2, HARS2, PARS2, GARS1, GATC, HARS1, IARS1, KARS1, MARS1, NARS1, YARS2, LARS1, GATB, SARS2, DALRD3, DARS2, QRSL1, IARS2, RARS2, VARS2, LRRC47, AARS2, QARS1, RARS1, SARS1, TARS1, VARS1, WARS1, CARS2, NARS2, TARS2, AARSD1, CARS1, YARS1, MARS2
AMINO ACID HOMEOSTASIS (GO:0080144)	SLC38A3, KCTD7, SLC7A11, GLS, GRM2, SLC66A1, SLC1A1, TPP2
AMINO ACID IMPORT ACROSS PLASMA MEMBRANE (GO:0089718)	ARL6IP5, SLC38A3, SLC6A14, SLC36A4, SLC43A2, AGT, CLN8, SLC36A1, TSPO2, ARL6IP1, SLC7A8, SLC7A11, SLC17A8, GFAP, GRM1, ITGB1, KCNJ10, ARG1, ATP1A2, NTSR1, SLC38A2, SLC6A20, SLC47A1, PSEN1, CLTRN, ACE2, RGS2, RGS4, SLC1A1, SLC1A2, SLC1A3, SLC1A4, SLC1A5, SLC1A6, SLC3A2, SLC6A6, SLC6A9, SLC6A13, SLC7A1, SLC7A2, SLC16A2, SLC22A2, SLC22A4, TNF, SLC7A5, SLC38A1, SLC7A3, SLC43A1, PER2, SLC6A5
AMINO ACID SALVAGE (GO:0043102)	BHMT2, MTAP, APIP, ADI1, ENOPH1, BHMT, MRI1
AMINO ACID TRANSMEMBRANE TRANSPORT (GO:0003333)	SLC25A13, SLC25A15, ARL6IP5, SLC38A3, SLC7A9, PRAF2, SLC6A14, SLC16A10, SFXN2, CLN3, SLC36A4, SLC15A4, SLC25A29, SLC38A10, SLC43A2, MFSD12, SLC32A1, SLC38A6, SLC38A8, SLC38A11, SLC66A1L, SLC38A9, SLC36A2, SLC7A13, AGT, CLN8, SLC36A1, TSPO2, ARL6IP1, SLC7A8, SLC7A11, SLC17A8, GFAP, SLC36A3, GRM1, SLC6A19, SLC6A18, ITGB1, KCNJ10, ARG1, SLC7A5P2, ATP1A2, NTSR1, SLC38A2, SLC6A20, SLC66A1, SLC25A38, SLC38A4, SLC6A15, LRRC8D, SLC38A7, SLC47A1, LRRC8A, SLC7A10, PSEN1, SLC17A7, SLC17A6, CLTRN, SLC7A14, ACE2, RGS2, RGS4, SLC1A1, SLC1A2, SLC1A3, SLC1A4, SLC1A5, SLC1A6, SLC1A7, SLC3A1, SLC3A2, SLC6A1, SLC6A6, SLC6A7, SLC6A9, SLC6A11, SLC6A12, SLC6A13, SLC7A1, SLC7A2, SLC7A4, SLC11A1, SLC16A2, SLC22A2, SLC22A4, TNF, SLC25A22, LRRC8E, SLC7A5, SLC38A1, SFXN3, SLC7A5P1, SLC25A18, SLC25A2, LRRC8C, SLC7A3, SLC43A1, SLC25A12, PER2, SLC7A7, SLC7A6, SLC6A5, SLC38A5, SFXN1
AMINO ACID TRANSPORT (GO:0006865)	SLC25A13, SLC25A15, ARL6IP5, PDPN, GIPC1, SERINC3, SLC38A3, SLC7A9, PRAF2, SLC6A14, HRH3, SLC16A10, SFXN2, SFXN4, CLN3, SLC36A4, SLC15A4, SLC25A29, SLC38A10, SLC43A2, MFSD12, ADORA1, ADORA2A, SLC32A1, SLC38A6, SLC38A8, CTNS, SLC38A11, SLC66A1L, SLC38A9, SLC36A2, SLC7A13, ABAT, AGT, CLN8, SLC36A1, TSPO2, RAB3GAP1, ARL6IP1, SLC7A8, SLC7A11, SH3BP4, SLC17A8, GABBR1, SERINC5, SLC17A5, GFAP, GJA1, SLC36A3, GRM1, GRM2, GRM7, APBA1, HTR1B, SLC6A19, SLC6A18, ITGB1, KCNJ10, ARG1, SLC7A5P2, SLC6A17, LEP, LLGL2, NF1, ATP1A2, NPY5R, NTRK2, NTSR1, OCA2, P2RX7, SLC38A2, SLC6A20, SLC66A1, SLC25A38, SLC38A4, AVP, SLC6A15, LRRC8D, AVPR1A, SLC38A7, SLC47A1, AVPR1B, LRRC8A, SLC7A10, PSEN1, SLC17A7, SLC17A6, CLTRN, SLC7A14, ACE2, RGS2, RGS4, SLC1A1, SLC1A2, SLC1A3, SLC1A4, SLC1A5, SLC1A6, SLC1A7, SLC3A1, SLC3A2, SLC6A1, SLC6A6, SLC6A7, SLC6A9, SLC6A11, SLC6A12, SLC6A13, SLC7A1, SLC7A2, SLC7A4, SLC11A1, SLC12A2, SLC16A2, SLC22A2, SLC22A4, SNCA, STXBP1, SYT4, TNF, TRH, TRPC4, TRPV1, XK, CACNB4, SLC25A22, LRRC8E, SLC7A5, SLC38A1, SFXN3, SLC7A5P1, SLC25A18, SLC25A2, DTNBP1, LRRC8C, SLC7A3, SLC43A1, KMO, SLC25A12, PER2, SLC7A7, SLC7A6, SLC6A5, SLC38A5, SLC9A3R1, SFXN1, SFXN5, SLC25A44, SV2A
C TERMINAL PROTEIN AMINO ACID MODIFICATION (GO:0018410)	ATG7, AGBL1, FOLH1B, AGTPBP1, FOLH1, ICMT, WIPI2, GPLD1, IRGM, LCMT1, ATG16L1, AGBL5, AGBL4, ATG12, ATG5
N TERMINAL PROTEIN AMINO ACID MODIFICATION (GO:0031365)	METAP2, NAA30, CREBBP, NTMT2, AANAT, EP300, METAP1, NAA80, METAP1D, NTMT1, NMT1, NAA20, PPM1A, PPM1B, HHAT, HHATL, NAA35, PDF, SOX4, NAA16, NAA40, NAA60, MAP6D1, NAA25, NAA15, NAA50, NAA10, NAA38, NAA11, KAT2B, NMT2
CELLULAR AMINO ACID BIOSYNTHETIC PROCESS (GO:0008652)	AASS, ENSG00000274276, SERINC3, SDS, ILVBL, PARK7, SDSL, CLN3, NOXRED1, CPS1, GOT1L1, CTH, NAGS, DHFR, ABAT, DPYD, AGXT, DHFR2, ALDH1A1, SEPHS2, SEPHS1, BHMT2, SERINC5, GAD1, GAD2, PHGDH, GGT1, GLS2, GLS, GLUD1, GLUD2, GLUL, GOT1, GOT2, PYCR2, PSAT1, ASL, ASNS, MTHFD2L, ASS1, MTAP, MTHFD1, MTHFR, MTR, MTRR, ATP2B4, OAT, OTC, PAH, PCBD1, APIP, LGSN, UPB1, PLOD2, HAO1, ASNSD1, ADI1, PSPH, DHFRP1, PYCR1, ALDH18A1, ENOPH1, BCAT1, BCAT2, AASDHPPT, BHMT, SRR, SHMT1,

	SHMT2, AGXT2, SLC1A3, PYCR3, CAD, SLC38A1, PCBD2, MRI1, SLC25A12, CBS, PSPHP1, PLOD3
CELLULAR AMINO ACID CATABOLIC PROCESS (GO:0009063)	AASS, RIDA, ENSG00000274276, BCKDK, CDO1, FTCD, SDS, HIBADH, HOGA1, SDSL, HYKK, AFMID, ACMSD, UROC1, ADHFE1, CARNMT1, CRYM, AMDHD1, TDH, DAO, DBT, IDO2, DLD, DLST, SARDH, ABAT, AGXT, ECHS1, AHCY, ETFA, ETFB, FAH, GCAT, HAAO, DDAH2, DDAH1, GAD1, GAD2, IL4I1, HIBCH, GCSH, ACAD8, GLS2, GLDC, GLS, GLUD1, GLUD2, AMT, GLUL, GOT1, GOT2, GPT, GSTZ1, HSD17B10, HAL, HDC, HGD, HMGCL, HNMT, HPD, ACADSB, IDO1, IVD, ACAT1, ARG1, ARG2, MIR21, MAT1A, ALDH6A1, ASPA, MTRR, NOS1, NOS2, NOS3, ATP2B4, OAT, OTC, PAH, AADAT, PIPOX, CSAD, HMGCLL1, AUH, ENOSF1, PRODH, KYAT3, MCCC1, CARNS1, PRODH2, QDPR, BCAT2, BCKDHA, BCKDHB, MCCC2, BLMH, ALDH8A1, SHMT1, SHMT2, AGXT2, TAT, TDO2, ALDH5A1, ASRGL1, GPT2, DDO, KMO, ALDH4A1, CBS, KYAT1, KYNU, ARHGAP11B, SLC25A21, SLC25A44
CELLULAR AMINO ACID METABOLIC PROCESS (GO:0006520)	GLYATL1B, FARSB, AASS, SLC25A13, RIDA, GLYAT, ENSG00000274276, BCKDK, WARS2, CDO1, MTHFS, FARS2, FTCD, SERINC3, SDS, ILVBL, HIBADH, HOGA1, PARK7, AZIN2, SDSL, CLN3, TPH2, NOXRED1, TARS3, HYKK, EARS2, AFMID, ACMSD, UROC1, AASDH, CPS1, GOT1L1, ADHFE1, CARNMT1, CRYM, TTC36, AMDHD1, PM20D1, CTH, CTNS, CTPS1, TDH, ADSS2, AARS1, DAO, DARS1, NAGS, DBT, DCT, DDC, IDO2, DHFR, DIO1, DLD, DLST, SARDH, ABAT, DPEP1, DPYD, AGXT, ECHS1, AHCY, DHFR2, EPRS1, ETFA, ETFB, ALDH1A1, FAH, FARSA, SEPHS2, SEPHS1, LARS2, SIRT4, HARS2, ICMT, GCAT, HAAO, FPGS, DDAH2, DDAH1, SLC7A11, BHMT2, SERINC5, GAD1, GAD2, IL4I1, PARS2, GARS1, GART, PHGDH, HIBCH, GCDH, GCSH, GFPT1, GGT1, GGT5, ACAD8, GLS2, GNMT, GCLC, GCLM, GLDC, GLS, GLUD1, GLUD2, AMT, GLUL, GOT1, GOT2, GATC, RIMKLA, GPT, GSS, GSTZ1, PYCR2, PSAT1, HSD17B10, HAL, HARS1, HDC, HGD, HMGCL, HNF4A, HNMT, HPD, IARS1, NAT8L, ACADSB, IDO1, INS, IVD, KARS1, ASPG, ACAT1, ARG1, ARG2, IYD, ACCSL, MIR21, MARS1, MAT1A, ART4, MECP2, ALDH6A1, ASL, MPST, ASNS, MTHFD2L, ASPA, ASS1, MSRA, MTAP, MTHFD1, MTHFR, MTR, MTRR, MMUT, NARS1, ATF4, NOS1, NOS2, NOS3, ATP2B4, OAT, ODC1, OTC, NOX4, PAH, PCBD1, PCCA, PCCB, YARS2, APIP, THAP4, AADAT, PIPOX, CSAD, LARS1, SCLY, LGSN, AZIN1, UPB1, PEPD, GATB, PFAS, PLOD2, ATP7A, HAO1, HMGCLL1, ASNSD1, PPAT, AUH, SARS2, DALRD3, DARS2, ADI1, THNSL2, QRSL1, ENOSF1, IARS2, PRODH, KYAT3, CTPS2, MCCC1, NIT2, BAAT, RARS2, VARS2, PSPH, DHFRP1, LRRC47, RIMKLB, AARS2, CARNS1, PTS, PYCR1, ALDH18A1, ENOPH1, PRODH2, QARS1, BCAT1, QDPR, BCAT2, RARS1, BCKDHA, BCKDHB, AASDHPPT, SARS1, BHMT, SRR, MCCC2, SLC39A8, FN3K, BLMH, ALDH8A1, SHMT1, SHMT2, AGXT2, SLC1A3, PYCR3, SLC16A2, SMS, BPHL, TARS1, TAT, TDO2, TH, TPH1, TST, TYR, VARS1, WARS1, CAD, ALDH5A1, CARS2, NARS2, AGMAT, DGLUCY, ASRGL1, TARS2, AARSD1, SLC38A1, CARS1, PCBD2, MRI1, ACCS, GPT2, HPDL, DDO, ATCAY, KMO, YARS1, SLC25A12, ALDH4A1, CBS, PSPHP1, KYAT1, GMPS, KYNU, ARHGAP11B, PLOD3, SLC25A21, SLC7A7, GLYATL1, MARS2, ACY1, SLC25A44, GFPT2, NR1H4
NEGATIVE REGULATION OF AMINO ACID TRANSPORT (GO:0051956)	ARL6IP5, HRH3, SLC43A2, ADORA1, ABAT, GABBR1, GRM7, HTR1B, LEP, NPY5R, RGS2, RGS4, TNF, TRH, SLC43A1
POSITIVE REGULATION OF AMINO ACID TRANSPORT (GO:0051957)	SLC38A3, ADORA2A, ABAT, AGT, RAB3GAP1, ARL6IP1, SLC17A8, GABBR1, ITGB1, NTSR1, P2RX7, AVP, AVPR1A, AVPR1B, PSEN1, CLTRN, ACE2, SLC6A1, SLC12A2, STXBP1, SYT4, TRH, SLC7A5, SLC38A1, DTNBP1, KMO
REGULATION OF AMINO ACID TRANSMEMBRANE TRANSPORT (GO:1903789)	ARL6IP5, SLC43A2, AGT, ARL6IP1, SLC17A8, ITGB1, ARG1, ATP1A2, PSEN1, CLTRN, ACE2, RGS2, RGS4, TNF, SLC7A5, SLC38A1, SLC43A1, PER2
REGULATION OF AMINO ACID TRANSPORT (GO:0051955)	ARL6IP5, SLC38A3, HRH3, SLC43A2, ADORA1, ADORA2A, ABAT, AGT, RAB3GAP1, ARL6IP1, SLC17A8, GABBR1, GRM2, GRM7, HTR1B, ITGB1, ARG1, LEP, ATP1A2, NPY5R, NTSR1, P2RX7, SLC38A2, AVP, AVPR1A, AVPR1B, PSEN1, CLTRN, ACE2, RGS2, RGS4, SLC6A1, SLC12A2, SNCA, STXBP1, SYT4, TNF, TRH, SLC7A5, SLC38A1, DTNBP1, SLC43A1, KMO, PER2, SV2A
REGULATION OF CELLULAR AMINO ACID METABOLIC PROCESS (GO:0006521)	BCKDK, PARK7, CLN3, ACMSD, SIRT4, SLC7A11, INS, MIR21, ATP2B4, BHMT, ATCAY, SLC7A7, NR1H4

RESPONSE TO AMINO ACID STARVATION (GO:1990928)	CDKN1A, RRAGB, NPRL2, RRAGA, GCN1, KPTN, ATF2, SESN3, KICS2, BMT2, DAP, EIF2S1, FLCN, RNF152, SZT2, LARP1, MTOR, RNF167, SESN1, HNRNPL, FAS, MAP3K5, EIF2AK4, ATF3, ATF4, SH3GLB1, LARS1, SLC38A2, MIOS, IMPACT, ITFG2, PRKD1, MAPK1, MAPK3, MAPK8, EIF2AK2, RRAGD, RRAGC, UCP2, TFEB, WDR59, NPRL3, SEH1L, SESN2, EIF2A, WDR24, MAP1LC3A, BECN1, DAPL1, EIF2AK3, DEPDC5

Table S2. Differentially expressed amino-acid metabolism-related genes between prostate cancer tumor and the non-tumor tissue with a threshold ∣log2FC∣≥0.585, and FDR<0.01.

Gene	logFC	FDR	Delta	Tumor (T)	Normal (N)	FC (T/N)
HAO1	3.992489	2.37E-08	189.4866	47.46076	2.865385	16.56349
NOX4	2.677369	2.47E-21	305.1447	113.9718	17.71154	6.434892
SLC6A19	2.675053	0.000127	932.1564	348.4628	54.48077	6.396069
DIO1	2.630937	4.34E-08	278.0694	105.6922	16.96154	6.231283
FOLH1B	2.399325	2.20E-08	2416.675	1007.231	190.8269	5.278246
CPS1	2.378665	1.03E-06	1145.105	481.4064	92.44231	5.207642
DPEP1	1.967645	0.000279	100.3657	51.00805	12.94231	3.941186
GLYATL1	1.900399	2.70E-14	7195.05	3786.072	1014.135	3.733304
SLC6A11	1.891221	7.57E-13	385.5123	203.8431	54.86538	3.715331
SLC7A11	1.876263	6.95E-22	1449.86	772.7384	210.4038	3.672644
SLC6A17	1.845875	8.27E-10	144.7842	78.43662	21.73077	3.609473
FOLH1	1.828613	2.08E-15	68162.39	37275.46	10494.52	3.551898
TDO2	1.787543	0.0044	979.5302	547.9759	158.6538	3.453908
SDS	1.727396	1.36E-14	281.3119	162.8531	49.09615	3.317024
ART4	1.711964	3.37E-06	74.57894	43.56338	13.21154	3.297374
GAD1	1.613835	4.07E-13	151.9148	94.1328	30.67308	3.068906
AGMAT	1.605656	7.41E-14	272.8388	169.9235	55.75	3.047956
SLC43A1	1.443293	2.92E-17	5767.286	3995.922	1469.346	2.719524
SLC3A1	1.438569	2.93E-05	229.4966	159.5312	58.76923	2.714536
SMS	1.36342	5.21E-11	32643.06	23942.04	9305.269	2.572955
PYCR1	1.33132	1.39E-40	13323.98	10008.1	3976.923	2.516543
MCCC2	1.294612	5.63E-12	33814.02	26119.03	10646.31	2.453342
SLC38A11	1.291759	1.02E-06	996.3386	771.3038	314.9038	2.449331
RIMKLA	1.184112	4.08E-22	741.4665	626.1791	275.5	2.272882
GRM7	1.18142	0.007213	122.0475	103.3058	45.48077	2.271418
CBS	1.154749	2.09E-14	5154.22	4463.497	2004.423	2.226824
SLC6A15	1.101115	0.003273	58.29706	52.94366	24.61538	2.150836
AADAT	1.006106	6.04E-12	1272.609	1264.885	629.7115	2.008674
SLC25A21	0.982167	5.81E-12	71.53616	72.83501	36.80769	1.978799
SFXN4	0.953356	1.14E-17	1847.647	1938.044	1000.654	1.936778
TMEM	0.952209	2.57E-11	120.1373	126.1670	65.13462	1.937019
MTHFD2L	0.952091	7.01E-21	1569.578	1648.559	852	1.934929
PPAT	0.904604	1.14E-22	835.7612	923.8974	493.3654	1.872643
MAP1D	0.895426	2.30E-21	213.4951	238.4286	128.0962	1.861325
PSAT1	0.868166	8.31E-12	1860.905	2143.491	1174.173	1.825532
GNMT	0.855411	0.000893	1868.296	2184.091	1207.192	1.809232
SOX4	0.852613	1.49E-13	4887.61	5732.509	3174.404	1.805854
IL4I1	0.850544	5.50E-10	142.2924	167.2958	92.71154	1.804476
SLC36A1	0.84678	3.82E-06	2918.716	3446.841	1916.135	1.798851
SLC25A22	0.843419	1.43E-24	2156.838	2557.258	1424.981	1.794591
SLC11A1	0.836854	2.91E-11	143.4791	171.4507	95.92308	1.787377
PTS	0.818708	7.67E-15	1026.154	1253.382	710.4808	1.764133
SFXN2	0.805263	3.89E-14	1302.369	1617.322	925.3846	1.747729
SLC25A15	0.799654	3.49E-14	652.558	816.0503	468.7308	1.740979
BPHL	0.795244	5.23E-16	955.8544	1201.964	692.5192	1.73564

BCAT1	0.773804	0.00045	257.2033	332.3883	194.3462	1.71029
ASNS	0.765823	3.43E-15	1348.964	1761.457	1035.635	1.700848
ACCS	0.762706	7.61E-11	605.8221	794.3058	468.0192	1.697165
MAP6D1	0.757655	4.99E-07	222.5418	293.7243	173.6731	1.691249
SHMT2	0.755294	7.99E-17	2325.826	3079.364	1824.135	1.688123
PYCRL	0.737696	4.22E-16	1381.386	1872.570	1122.731	1.667870
ACAD8	0.737344	2.96E-05	4479.9	6075.728	3644.212	1.667227
ODC1	0.722898	7.90E-07	15323.22	21196.95	12841.81	1.65062
MPST	0.713903	2.05E-12	2514.76	3522.549	2147.404	1.640376
AGPHD1	0.711082	3.75E-12	131.2354	184.5573	112.6731	1.637990
MARS2	0.698331	2.05E-10	318.0624	455.4608	280.5962	1.62319
GCSH	0.693887	1.98E-05	64.04562	92.2998	57	1.619295
NAA38	0.693339	1.62E-12	918.6751	1325.002	819.25	1.617335
CSAD	0.690101	9.44E-07	799.5538	1158.604	717.9615	1.613741
SNCA	0.687832	0.000294	521.8151	758.6378	470.8846	1.611091
KPTN	0.678962	1.02E-15	302.7092	445.841	278.4038	1.601418
ACY1	0.659374	3.62E-13	1913.453	2901.922	1837.077	1.579641
NIT2	0.658105	4.31E-15	1392.017	2115.191	1340.173	1.578297
C19ORF28	0.646234	1.05E-17	2028.147	3138.409	2004.827	1.565426
SLC16A10	0.641675	0.000213	73.09154	113.9074	72.96154	1.561199
SERINC5	0.626187	0.002294	1203.111	1921.328	1244.673	1.543641
ASRGL1	0.616111	1.00E-04	1754.794	2848.179	1858.019	1.532912
MIOS	0.61422	2.59E-18	2155.681	3509.624	2292.558	1.530877
SLC7A1	0.605709	9.93E-08	3146.894	5195.386	3413.019	1.522226
APBA1	0.601756	5.28E-08	310.3039	515.664	339.7115	1.517947
SDSL	0.600362	3.11E-10	636.0475	1059.441	698.6538	1.516403
IDO1	0.596046	0.009556	160.701	269.6117	178.25	1.512548
BCAT2	0.595139	3.01E-12	2275.255	3823.066	2530.481	1.510806
GSTZ1	0.591005	1.98E-09	1336.245	2260.97	1500.885	1.506425
UCP2	-0.60329	4.24E-05	-896.934	1486.726	2257.904	0.658454
SLC6A1	-0.62326	3.31E-05	-20.3758	32.69215	50.38462	0.648852
CDKN1A	-0.63026	2.07E-05	-2801.69	4445.326	6879.769	0.646145
SLC38A1	-0.63895	9.92E-14	-3595.78	5627.614	8759.462	0.642461
ILVBL	-0.64221	2.01E-29	-1636.93	2548.895	3977.327	0.640856
SLC6A6	-0.64427	1.32E-09	-458.036	710.9356	1110.904	0.639961
P2RX7	-0.6468	9.97E-06	-43.0438	66.5493	104.25	0.638363
SLC25A12	-0.65067	3.29E-24	-832.187	1278.97	2007.596	0.637065
GLUL	-0.65133	7.49E-16	-10752.1	16507.91	25920.6	0.636864
ITGB1	-0.67087	7.83E-27	-13028.1	19419.78	30907.42	0.628321
PER2	-0.72462	1.42E-12	-1080.37	1490.952	2463.365	0.60525
SLC16A2	-0.79804	1.00E-17	-560.675	702.5674	1221.538	0.57515
RNF152	-0.80113	5.87E-12	-54.0724	67.49497	117.6731	0.57358
DPYD	-0.80515	8.21E-12	-587.991	730.2877	1275.923	0.57236
HPDL	-0.80513	4.00E-05	-31.5652	39.20523	68.59615	0.571537
SLC1A3	-0.85388	1.63E-12	-301.516	353.1127	638.0769	0.553401
OAT	-0.86952	2.41E-15	-3193.58	3672.825	6708	0.547529
SV2A	-0.87504	5.88E-15	-188.409	215.3159	394.9231	0.54521
SFXN3	-0.90657	2.19E-25	-749.858	827.1388	1550.135	0.533592
ATF3	-0.9081	9.08E-05	-5506.74	6064.004	11379.02	0.532911
SLC7A4	-0.90881	0.001077	-318.298	350.2374	657.4423	0.532727
C14ORF159	-0.92999	2.92E-18	-1271.09	1366.779	2603.461	0.524985
AMT	-0.93553	7.11E-13	-513.458	548.839	1049.577	0.522915
PDPN	-0.97944	5.56E-17	-652.918	666.6258	1314.231	0.507236
DDO	-1.00969	1.01E-15	-73.2746	72.57143	146.2115	0.496345

TRPC4	-1.04722	2.66E-17	-211.803	202.2535	418	0.48386
NTRK2	-1.09521	5.45E-14	-517.542	472.5493	1009.308	0.468192
SLC38A5	-1.14686	3.30E-16	-120.481	105.0523	232.6731	0.451502
HAAO	-1.15788	2.36E-19	-295.048	254.8169	568.6538	0.448105
GPLD1	-1.17097	2.36E-19	-66.8489	57.08853	128.6538	0.443737
GJA1	-1.24716	2.75E-22	-5279.45	4233.181	10046.4	0.421363
ASS1	-1.29312	1.12E-21	-4294.61	3321.123	8134.25	0.408289
ATCAY	-1.30812	1.39E-14	-204.393	156.2495	387	0.403745
SLC47A1	-1.35313	6.09E-29	-172.992	127.8451	326.7115	0.391309
PIPOX	-1.36382	3.84E-24	-111.968	82.09859	211.4423	0.388279
BHMT2	-1.45812	2.32E-33	-331.445	227.3099	624.6154	0.36392
ATP2B4	-1.47074	1.86E-31	-6832.02	4645.292	12871.37	0.360901
NAGS	-1.47573	2.02E-49	-132.46	89.75855	249.6731	0.359504
PRODH	-1.53445	1.70E-24	-122.071	79.55332	230.6346	0.344932
HMGCLL1	-1.65345	5.18E-15	-68.1006	41.18712	129.8269	0.317246
ATP1A2	-1.66926	1.36E-20	-2347.11	1406.082	4471.731	0.314438
SLC7A5	-1.67777	2.00E-26	-1250.62	745.4044	2382.865	0.312819
DHDPSL	-1.70902	9.64E-24	-55.7270	32.60765	106.8462	0.305183
NOS1	-1.855	2.12E-14	-72.3561	39.00604	141.4231	0.275811
ASPA	-2.05455	6.87E-31	-73.6951	35.86922	149.3654	0.240144
CDO1	-2.23805	7.04E-28	-1325.97	592.4688	2791.942	0.212207
ACE2	-3.7763	6.33E-99	-109.186	28.91348	397.0385	0.072823
